# Chemical composition and anti-oxidant potential on essential oils of *Thymus quinquecostatus* Celak. from Loess Plateau in China, regulating Nrf2/Keap1 signaling pathway in zebrafish

**DOI:** 10.1038/s41598-020-68188-8

**Published:** 2020-07-09

**Authors:** Ting He, Xiao Li, Xiaoping Wang, Xiao Xu, Xin Yan, Xiang Li, Siqi Sun, Ying Dong, Xueyang Ren, Xiaoyun Liu, Yu Wang, Hong Sui, Qing Xia, Gaimei She

**Affiliations:** 1 School of Chinese Materia Medica, Beijing University of Chinese Medicine, Beijing, 102488 China; 2 School of Chinese Pharmacy, Ningxia Medical University, Yinchuan, 750004 China; 3 Biology Institute, Qilu University of Technology (Shandong Academy of Sciences), Jinan, 250103 China

**Keywords:** Chemical biology, Molecular biology, Plant sciences, Health care, Chemistry

## Abstract

Chemical profile and antioxidant potency of essential oils (EOs) of *Thymus quinquecostatus* Celak. (thyme oils) obtained from Loess Plateau in China had been studied. 130 constituents of thyme oils were determined using gas chromatography-mass spectrometry (GC–MS) and carvacrol ethyl ether was firstly reported as a new natural product, which has been used as a synthetic flavoring substance with no safety concern. The thyme oils showed the anti-oxidant activity using 2,2 diphenyl-1-picrylhydrazyl (DPPH), 2,2′-azino-bis-(3-ethylbenzothiazoline-6-sulfonate) (ABTS), ferric reducing antioxidant power (FRAP) and thiobarbituric acid reactive substances (TBARS) and conferred protection against oxidative stress in zebrafish. In addition, a class of carvacrol analogues was found to develop as potential natural antioxidant products of thyme oils from Loess Plateau by the correlation analysis. YL-thyme oil performed the best antioxidant activity in this research, which could be recommended as preferred sources of thyme oils. Furthermore, YL-thyme oil exhibited a potent antioxidant capacity by reactive oxygen species (ROS) scavenging, enhancing the endogenous antioxidant system, inhibiting lipid peroxidation and activation of Keap1/Nrf2 pathway in zebrafish.

## Introduction

Oxidative stress is a phenomenon associated with pathogenetic mechanisms of several diseases including atherosclerosis, neurodegenerative diseases, cancer, diabetes mellitus, inflammatory diseases, as well as psychological diseases or aging processes. It is defined as an imbalance between production of free radicals and reactive metabolites, so-called oxidants or ROS^1^. Most of ROS such as superoxide anion (O^2−^), hydroxyl radical (OH^·^), hydrogen peroxide (H_2_O_2_) and organic peroxides, are generated in the cells by mitochondrial respiratory chain^2^. Excessive ROS can damage cell functionality as they can harm cellular lipids, proteins and DNA^3^. The organism contains a complex and carefully balanced cascade of antioxidant enzymes, of which the superoxide dismutase (SOD) and catalase (CAT) are regarded as the first line of the antioxidative enzyme system against ROS such as superoxide and H_2_O_2_ generated during oxidative stress^4^. The kelch-like ECH-associated protein 1/nuclear factor E2-related factor 2 (Keap1/Nrf2) signaling pathway plays a central role in protecting the body from oxidative damage. The Nrf2 transcription factor is tightly regulated by the repressor protein, Keap1, in the cytoplasm. Under oxidative stress, Nrf2 is released from the Nrf2/Keap1 complex and is translocated to the nucleus. Subsequently, it binds to antioxidant-response elements (AREs) and upregulates the expression of downstream genes, such as Sod1, Cat and Hmox1, which in turn, activates cellular antioxidant defensive capacity^5^.

The beneficial effects of antioxidants on the maintenance of health in human have become an important subject that has engaged many scientists across the world over the last decade. The antioxidants from plants have been reported to inhibit the propagation of free radical reactions and protect the human body from various diseases^6^. The genus *Thymus* belongs to the Lamiaceae family, and contains more than 300 species of aromatic perennial herbaceous plants with valuable medicinal properties^7^. *T*. *quinquecostatus*, commonly called thyme (‘thyme’ in this study refers to *T*. *quinquecostatus*), was distributed in the northwest of China, mainly in the Loess Plateau. It is locally used as a seasoning and also has natural preservative effects which makes it widely used as a green harmless flavor additive. Thyme was widely used to treat stroke, cold, dyspepsia, toothache, acute gastroenteritis, hypertension, chronic eczema and other diseases as folk medicines and was used alone or in combination with other herbal medicines for cancer treatment. In our previous researches, the antioxidant activities of different polar fractions of *T*. *quinquecostatus* had been evaluated. Meanwhile, chemical compositions, like flavonoids and phenolic acids, were also identified and full-scale qualitative and quantitative methods were successfully applied to the quality evaluation of *T*. *quinquecostatus*^8,9^.

Thyme oil has been widely used in food industry, pharmaceutical and cosmetic due to its various biological activities, including antioxidant, anti-inflammatory, antitumor and antimicrobial effects^10^. To our best knowledge, remarkable chemo diversity of the same plant species has been reported due to the differences in chemotype, plant genotype, harvesting time, cultivation and climatic peculiarities and other factors; therefore, investigation of the same plant species from different geographic zones may considerably expand the existing knowledge on essential oils (EOs).

And previous reports mainly focused on thyme oils from one certain district, while the diversity of chemical compositions in thyme oils collected from different producing areas has not yet been studied and the relationship between thyme oils compositions and biological activities is rarely mentioned. Moreover, most of the antioxidant researches on thyme oils focused on in vitro studies and the underlying mechanism of antioxidant activity of thyme oils is still unclear.

**Figure 1:**
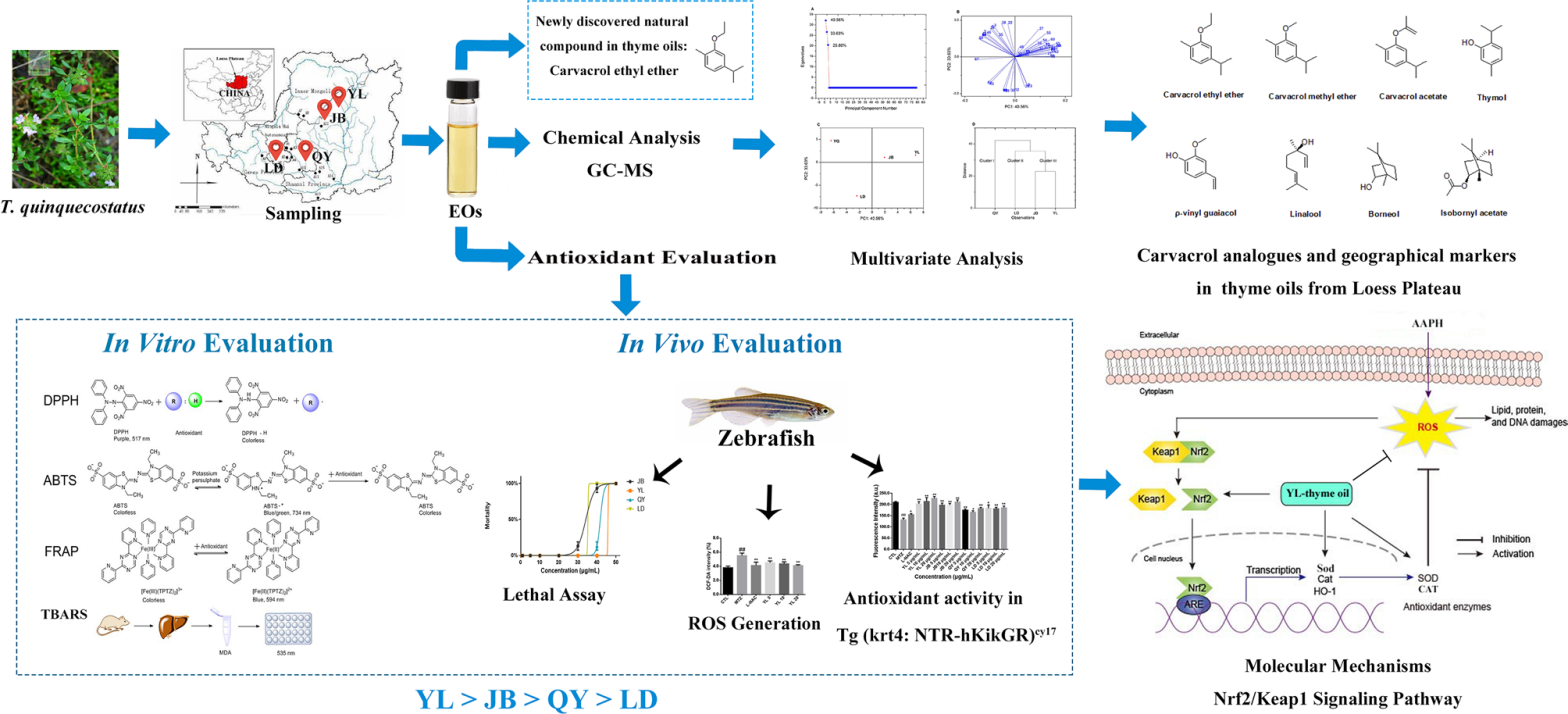
Graphical abstract.

**Figure 2:**
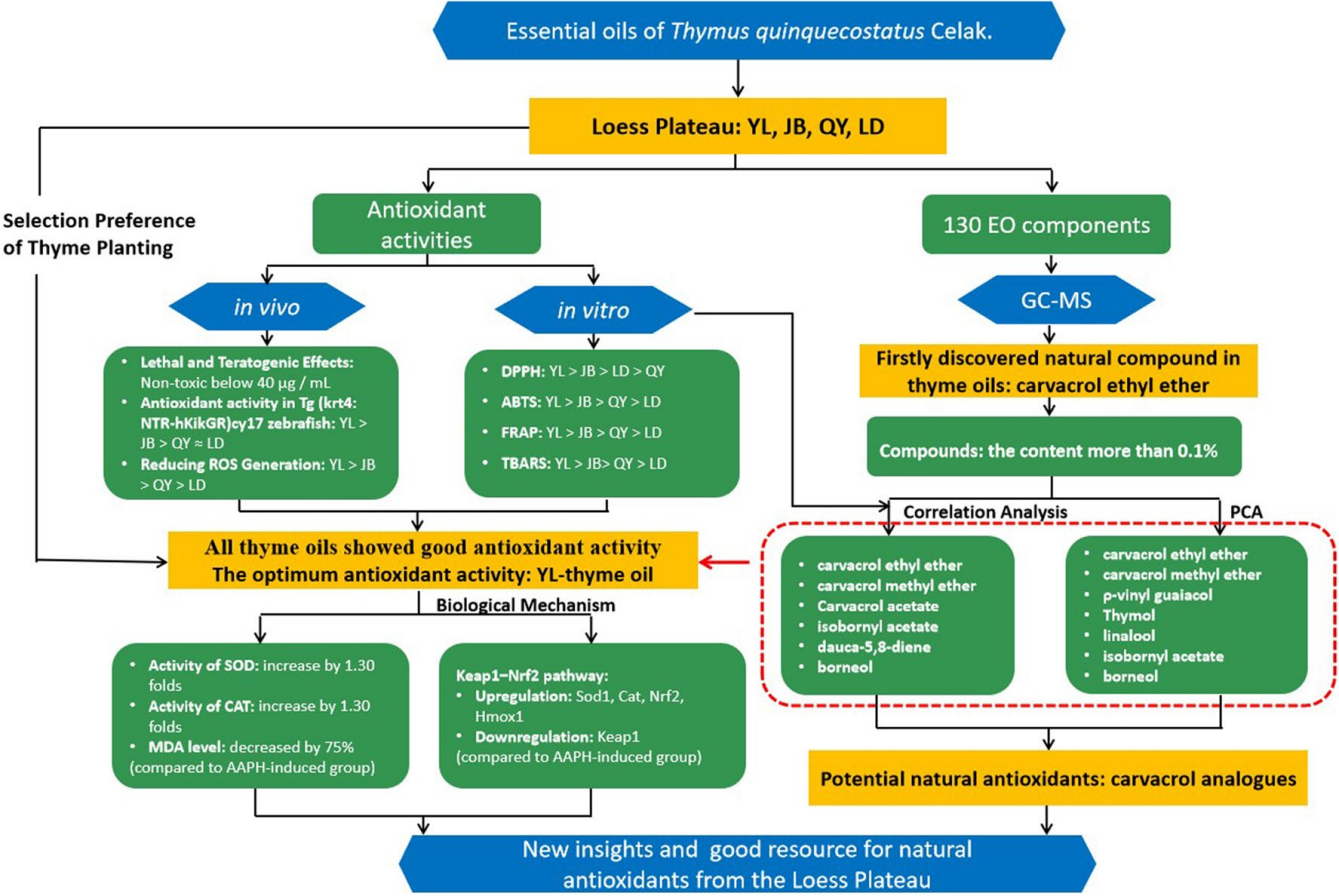
Technical route.

Considering polymorphism of *Thymus* genus plants as well as the variety of factors, which may influence EO compositions and their biological activities, *T*. *quinquecostatus* cultivated in the Loess Plateau of four main producing regions, namely YL (Shaanxi province), JB (Shaanxi province), QY (Gansu province) and LD (Ningxia Hui Autonomous Region) had been selected for the present study. Combined with statistical analysis, the compositions of thyme oils from the four producing areas were comparatively analyzed and the correlations between these thyme oil components and anti-oxidative activities were also discussed. Besides, their antioxidant potentials were evaluated by the in vitro assays (DPPH, ABTS, FRAP and TBARS) as well as in the in vivo zebrafish model. Moreover, the effects of thyme oils on expression of genes related to oxidative stress were investigated. This study provides a better understanding of antioxidant capacities of thyme oils and the underlying molecular mechanisms. Figures 1 and 2 are graphical abstract and technical route of this study, respectively.

## Materials and methods

### Plant materials

The aerial parts of *T*. *quinquecostatus* were collected from YL (38°17′4.89″N, 109°43′44.43″E) and JB (37°35′55.44″N, 108°47′26.40″E) in Shaanxi province, QY (35°42′36.62″N, 107°38′17.42″E) in Gansu province and LD (35°37′5.75″N, 106°07′8.27″E) in Ningxia Hui Autonomous Region in the northwest of China in the month of August. They were carefully identified by Dr. Shengjun Ma (Beijing University of Chinese Medicine). Those samples were separately dried in shade and deposited at the Lab of Engineering Research Center of GAP for Chinese Crude Drugs, Ministry of Education, Beijing University of Chinese Medicine.

### Extraction of Eos

Dry samples (300 g) of thyme from the four producing areas were severally soaked in deionized water at room temperature about 25 °C to stand overnight, and then were subjected to hydrodistillation in a modified clevenger-type apparatus for 10 h. The obtained EOs were extracted with *n*-hexane. Subsequently, the extracts were exsiccated by anhydrous sodium sulphate and stored in a stopper vials at 4 °C in a refrigerator until the time of analysis.

### GC–MS analysis

EOs were analyzed by an HP 5890 gas chromatograph (GC) using an HP 5973 mass selective detector (Finnigan Trace-MS 2000, USA) with the electron ionization mode (70 eV). The capillary column was a HP-5MS (5% phenyl methyl siloxane, 30 m × 0.25 mm i.d., 0.25 μm film thickness). The temperature of the inlet and detector, injection volume and split ratio were set to 260 °C, 260 °C, 1 µL (TBME solution), 40 : 1, respectively. The oven temperature started at 50 °C, ramped to 100 °C at a rate of 5 °C/min, ramped to 150 °C at a rate of 3 °C/min, and ramped to 250 °C at a rate of 5 °C/min, then held at 250 °C for 30 min. Helium (99.999%) carrier gas was kept with a constant flow of 1.2 mL/min. A standard n-alkane mixture (C_6_–C_24_) was also analyzed using the above conditions to determine the retention index (RI).

### Identification of compounds

The thyme oils were identified by the retention indices (RI), relative to the series of alkanes (C_6_–C_24_) at the same chromatographic conditions, referring to the Van Den Dool method^11^. The data were analyzed by the Xcalibur 1.1 software, compared with NIST/EPA/NIH database (1998, version 1.6) and several references^12,13^. The book “*Identification of Essential Oils Components by Gas Chromatogrtaphy/Mass Spectrometry*, *4th edition*” (Robert P. Adams, PhD) equally played a vital role in identifying the individual compounds.

### DPPH assay

The DPPH test was carried out as described before^14^. 0.1 mL thyme oils at various concentrations of (0.02–5.67 mg/mL) were added in 0.1 mL ethanol solution of DPPH (0.001 mg/mL) acquired from Sigma-Aldrich (Saint Louis, MO, USA). The absorbance of each solution was measured at 517 nm, with ethanol being a blank. Ascorbic acid was used as a positive control. Simple regression analysis was used to derive the IC_50_ value.

### ABTS assay

The antioxidant activity of the samples was determined by using the method described previously^15^. An ABTS stock solution was prepared by dissolving ABTS (Sigma-Aldrich) (7 mM) in water. An ABTS radical cation (ABTS^·+^) solution was then prepared by mixing the ABTS stock solution with potassium persulfate solution (2 mM) in equal quantities and in the dark at room temperature. The ABTS^·+^ solution was diluted with PBS (10 mM, pH 7.4) to an absorbance of 0.70 ± 0.02 at 734 nm and stabilized at 30 °C. Next, 0.1 mL thyme oils at different concentrations (0.02–2.32 mg/mL) were mixed with 0.1 mL dilution ABTS^•+^ solution with ethanol; after 10 min, the absorbance of the mixture was measured at 734 nm in the dark at room temperature. As a positive control, ascorbic acid was chosen to be employed. Simple regression analysis was used to derive the IC_50_ value.

### FRAP assay

FRAP activity was estimated by the assay described by Benzie and Strain (1996)^16^. 5 mL of freshly prepared FRAP reagent and 20 uL thyme oils (2.32–16.87 mg/mL) were mixed and the resulting solution was vortexed and then incubated at room temperature for 70 min. After the incubation, the absorbance was measured at 594 nm. Ferrous sulfate solutions (FeSO_4_) (1–33 mmol·L^−1^) were used to obtain the standard curve and trolox (0.975 mg/mL) was used as the positive control. The results were expressed as mmol of ferrous sulfate equivalents per gram per liter of thyme oils. The ferric reducing activity of all samples was expressed as FeSO_4_ equivalent (mmol·L^−1^·g^−1^DW).

### Measurement of lipid peroxidation by thiobarbituric acid reactive substances (TBARS) assay

The TBARS assay is widely used to measure lipid oxidation and antioxidant activity in food and physiological systems. Modified TBARS were used to measure the lipid peroxide using mouse liver homogenates as lipid-rich media^17^. Briefly, thiobarbituric acid reacts with malondialdehyde (MDA) to form a diadduct, a pink chromogen, which can be detected spectrophotometrically at 535 nm. 0.2 mL thyme oils at different concentrations (0.18–1.82 mg/mL) were mixed with 10% liver homogenate (each 100 mL homogenate solution contains 10.0 g mouse liver), then 0.1 mL of FeSO_4_ · 7H_2_O (5 mM) and 0.1 mL H_2_O_2_ (100 mM) were added to initiate lipid peroxidation. After incubation for 60 min at 37 °C, the reaction was stopped by addition of 0.3 mL 0.68% thiobarbituric acid in TCA-HCl (16.8% w/v trichloroacetic acid in 0.125 N HCl). The reaction mixtures were heated for 60 min at 95 °C. The samples were cooled and centrifuged, and the absorbance of the supernatants was measured at 535 nm. Simple regression analysis was used to derive the IC_50_ value. The results were compared to that of ascorbic acid employed as the reference.

### Zebrafish maintenance

The adult zebrafish AB strain and Tg (krt4:NTR-hKikGR)^cy17^ transgenic zebrafish lines were obtained from Drug Screening Platform of Shandong Academy of Sciences (Jinan, Shandong, China). The adult zebrafish used in this study were reared at 28 ± 0.5 °C with a 14 : 10 h light–dark cycle in an automatic zebrafish housing system (ESEN, Beijing, China) with fish water (5 mM NaCl, 0.17 mM KCl, 0.4 mM CaCl_2_, and 0.16 mM MgSO_4_). Embryos were collected and maintained in the zebrafish spawning box after natural spawning induced by light in the morning and then maintained in a light incubator at 28 °C until 24 h post fertilization (hpf). At the end of the experiments, all zebrafish were euthanized with an overdose of tricaine (200 mg/L).

### Lethal and teratogenic assay

As depicted in previous research^18^, normal developing embryos were selected under a stereomicroscope (SZX16, Olympus, Tokyo, Japan) at 24 hpf and were randomly tranferred to 24-well plates at a density of 15 per well. Thyme oils were diluted with fish water to different concentrations (1, 5, 10, 20, 40 μg/mL) in each well to a final volume of 2 mL. Mortality rates were investigated till 48 hpf. Also, morphological changes of craniofacial features, brain, kidney, yolk sac, swim bladder, pericardium and body shape were monitored under the stereomicroscope. And the value of 10% lethal concentration (LC_10_) and 1% lethal concentration (LC_1_) were calculated. In subsequent experiments, thyme oils were used at concentrations of 1/3 LC_1_, LC_1_, LC_10_.

### Antioxidant activity in Tg (krt4: NTR-hKikGR)^cy17^ zebrafish larvae

24-hpf Tg (krt4: NTR-hKikGR)^cy17^ zebrafish (15 embryos/well) were added to 24-well plates, and incubated with or without metronidazole (MTZ) (5 mM) (Sigma-Aldrich). Thyme oils were diluted with fish water to different concentrations (5, 10 and 20 μg/mL) to a final volume of 2 mL. After 24 h of incubation, zebrafish larvae were anesthetized using 0.16% tricaine, and then fluorescence was detected by FSX100 Bio Imaging Navigator Equipment. N-acetyl-l-cysteine (L-NAC) (0.2 mM) was used as a positive control. L-NAC is a classical antioxidant that can reduce the damage to cells caused by oxidative stress and has been widely used as an antioxidant positive control *in vivo*^19,20^. The relative antioxidant capacity of the thyme oils can be determined by counting the number of fluorescence spots using Image—Pro Plus software. A fixed square region (18.00 cm × 6.00 cm) of the images on zebrafish body was selected and calculated the number of fluorescence.

### Measurement of intracellular ROS production in AB zebrafish larvae

The dichloro-dihydro-fluorescein diacetate (DCFH-DA) method was used to quantify intracellular ROS levels^21^. All treatment solutions were diluted to different concentrations using fish water. 24-hpf AB zebrafish (15 embryos/well) were added to 24-well plates, and incubated with or without thyme oils (5, 10 and 20 μg/mL) to a final volume of 2 mL for 1 h. Then, 2,2-Azobis (2-amidinopropane) dihydrochloride (AAPH) (12 mM) was added to incubate for 24 h. L-NAC (0.2 mM) was used as a positive control. A ROS kit (Nanjing Jiancheng Bioengineering Institute, Nanjing, China) was used for analyzing generation of ROS in larvae at 48 hpf. The treatment groups were treated with 30 μM DCFH-DA and incubated in the dark at 28 ± 0.5 °C for 40 min. The zebrafish larvae were then washed with fish water three times and were observed using a fluorescence microscope (SZX16, Olympus, Tokyo, Japan). Fluorescence intensity of each zebrafish larva was determined using the Image—Pro Plus software.

### Assessment of antioxidative enzyme and lipid peroxidation activities in AB zebrafish larvae

The test was carried out as described before^18^. Briefly, 24-hpf AB zebrafish were transferred into six-well plates (100 larvae/3 well/group), and incubated with or without AAPH (12 mM) and YL-thyme oils (5, 10 and 20 μg/mL) to a final volume of 5 mL. L-NAC (0.2 mM) was used as a positive control. All treatment solutions were diluted to final different concentrations using fish water. Larvae in each group were pooled together and homogenized on ice with 500 μL ice-cold physiological saline at 48 hpf. The supernatants were collected for analysis of antioxidative enzyme and lipid peroxidation activities after centrifugation at 3,500 rpm for 15 min at 4 °C. SOD, CAT activity and MDA levels were assessed using commercial kits (Nanjing Jiancheng Bioengineering Institute) in accordance with the manufacturer’s protocols. SOD and CAT activity were expressed as unit per milligram protein and MDA levels were showed as nmol per milligram protein. And the protein content was determined using the bicinchoninic acid method^22^.

### Quantitative real-time polymerase chain reaction (qRT-PCR) assay in AB zebrafish larvae

24-hpf AB zebrafish were transferred into six-well plates (30 embryos/well), and incubated with or without AAPH (12 mM) and YL-thyme oils (5, 10 and 20 μg/mL) to a final volume of 5 mL. L-NAC (0.2 mM) was used as a positive control. All treatment solutions were diluted to final different concentrations using fish water. Total zebrafish RNA was extracted from 30 larvae (48 hpf) using the TRIzol reagent (Invitrogen, Waltham, USA) following the manufacturer’s instructions. RNA concentrations and quality were evaluated on the basis of OD_260_/OD_280_ ratio. Then the synthesis of cDNA was carried out by using the HiScript II Q RT SuperMix (Vazyme, Nanjing, China), and RT-PCR was done by using a RT-PCR system (Biorad, CA, USA) using the SYBR Green mix (Takara, Dalian, China). Thermal cycling was set at 95 °C for 5 s and 60 °C for 30 s with 40 cycles. Transcription of target genes was calculated using the 2^−ΔΔCt^ method. *β-actin* was chosen as the house-keeping gene. The primer sequences of the genes that were detected are shown in Table 1.

**Table 1:** The sequences of primer pairs used in real-time quantitative PCR assay.

No	Gene symbol	Forward primer (5′–3′)	Reverse primer (5′–3′)
1	Sod1	ATGGTGAACAAGGCCGTTTG	CATGAGGGTTGAAGTGCGGA
2	Cat	ACTACAAGACTAATCAGGGCATTAAGAA	CCCACAGGAATCAGAGGGAACT
3	Keap1	CCAACGGCATAGAGGTAGTTAT	CCTGTATGTGGTAGGAGGGTT
4	Nrf2	TTGTCTTTGGTGAACGGAGGT	CTCGGAGGAGATGGAAGGAAG
5	Hmox1	ATGCCCTTGTTTCCAGTCAGC	GGACTTGGAGCACTTCTTCGG

### Statistical analysis

All the assays were conducted in triplicate and results were expressed as mean values ± standard deviation (SD). Analysis of variance (ANOVA) test was performed to check significant differences. Comparison between the groups was performed using Student–Newman–Keuls (SNK). Differences from controls were considered significant when *p* was less than 0.05 or 0.01. Removal of the compounds with concentrations less than 0.1% had no effect on the overall analysis. Therefore, the thyme volatile constituents with the content more than 0.1% were evaluated using Principal Component Analysis (PCA) and Hierarchical Clustering Analysis (HCA), which could highlight the similarity and dissimilarity of the EO constituents and expound the active ingredients of thyme oils. Correlation analyses were performed by using a two-tailed Pearson’s correlation test. All statistical analyses were carried out by using OriginPro 2017 and GraphPad Prism (version 6.01).

## Results and discussions

### Chemical compositions of thyme oils

**Figure 3:**
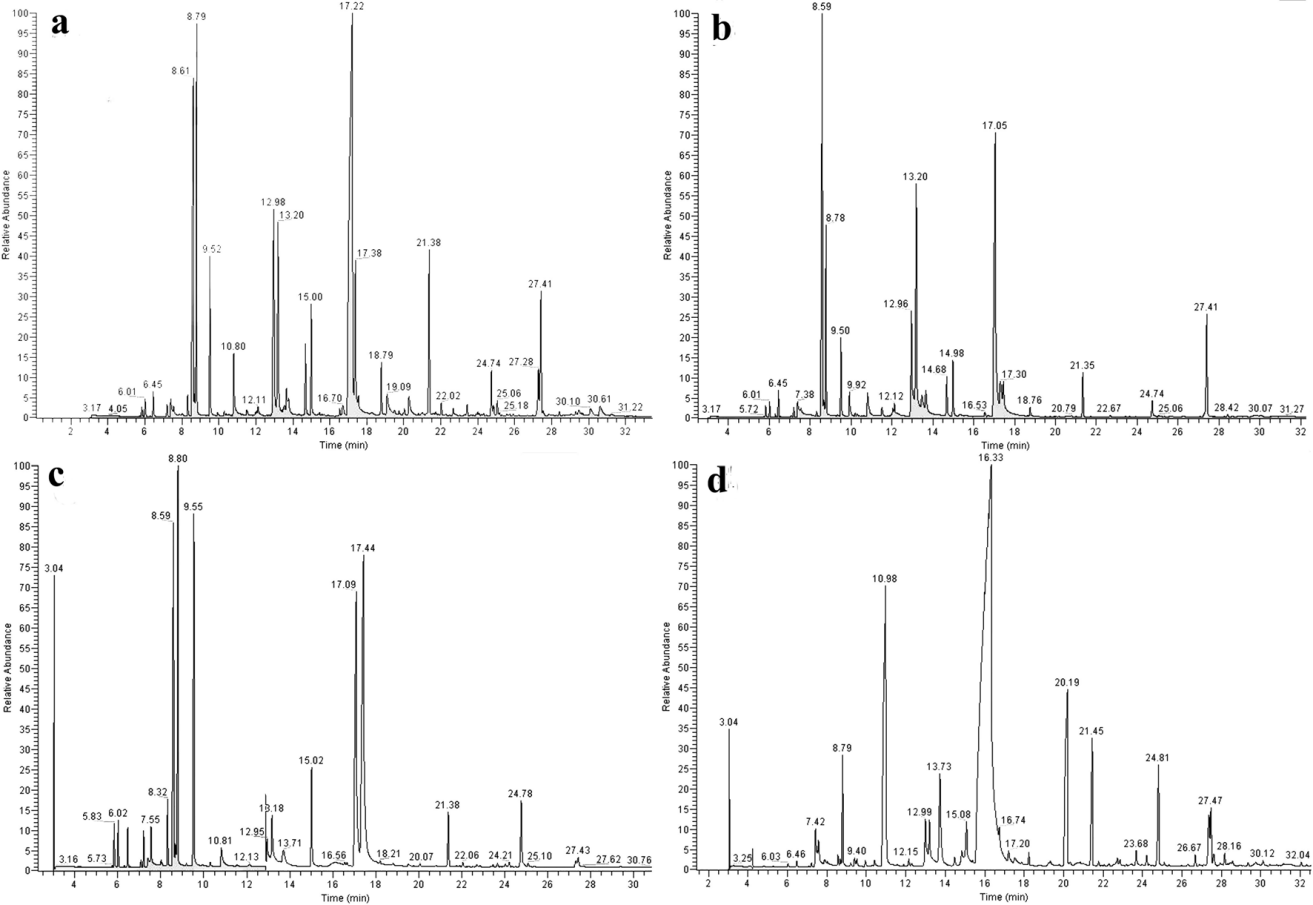
Total ion chromatograms (TICs) given by GC–MS of the YL-thyme oil (**a**), JB-thyme oil (**b**), QY-thyme oil (**c**) and LD-thyme oil (**d**).

The aerial parts of *T*. *quinquecostatus* were hydrodistilled and produced a yellow EOs with characteristic odor, with the yield of 4.67 mL/kg (YL), 2.33 mL/kg (JB), 5.00 mL/kg (QY) and 2.67 mL/kg (LD). Total ion chromatograms (TICs) given by GC–MS of the thyme oils was shown in Fig. 3 (YL-thyme oil (Fig. 3a), JB-thyme oil (Fig. 3b), QY-thyme oil (Fig. 3c) and LD-thyme oil (Fig. 3d)). And the retention indices (RI) and percentages of thyme oil chemical compositions were listed in Table 2. Monoterpenes are the most frequent metabolites of the thyme oils, including hydrocarbon monoterpenes (1.29–29.88%), oxygenated monoterpenes (17.09–27.89%) and phenolic monoterpenes (0.38–16.32%).

**Table 2:** Chemical constituents of the thyme oil in the four regions. a: “–” means the compound is not identified; b: “tr” represent the percent of compound is lower than 0.05%; c:“RI” represent the Retention indices on the DB-5 column.

No	Name	RI	Samples			
			QY	LD	JB	YL
Those composition possessing more than 0.1%						
1	Artemisia triene	923	–	–	–	0.20
2	α-Thujene	926	1.01	–	0.36	–
3	α-Pinene	933	1.04	0.07	0.46	0.38
4	α-Fenchene	945	0.89	0.12	–	–
5	Camphene	949	–	–	0.81	0.50
6	Artemiseole	973	0.18	–	–	–
7	1-Octen-3-ol	974	–	–	1.15	
8	β-Pinene	978	0.84	tr	0.30	0.25
9	3-Octanone	979	–	0.84	–	0.59
10	1-Decene		0.41	–	–	–
11	6-Methyl-5-hepten-2-one	981	–	0.26	–	–
12	3-Octanol	988	–	0.12		tr
13	Myrcene	990	–	0.64	0.54	0.27
14	Methyl-pentanoatel(2-Hydroxy-3-methyl-)	991	1.20	–	–	–
15	*iso*-Sylvestrene	1007	0.13	–	–	–
16	α-Terpinne	1014	–	–	–	0.46
17	α-Terpinene	1017	1.62	–	0.20	–
18	ρ-Cymene	1020	–	0.18	19.2	–
19	*ο*-Cymene	1022	12.10	–	–	–
20	(*Z*)-β-Ocimene	1031	0.60	–	–	–
21	Limonene	1024	–	0.13	0.69	0.40
22	1,8-Cineole	1026	10.16	1.84	5.22	7.23
23	Prenyl isobutyrate	1048	–	0.23	–	–
24	γ-Terpinene	1054	11.11	0.15	2.73	3.03
25	*cis*-Sabinene hydrate	1065	–	–	1.28	–
26	*cis*-Linalool oxide	1067	–	0.13	–	0.08
27	Terpinolene	1086	0.13	–	0.10	0.21
28	Linalool	1095	1.22	12.80	1.92	1.80
29	*cis*-ρ-Menth-2-en-1-ol	1118	–	–	0.58	–
30	Camphor	1141	0.11	0.12	0.63	–
31	*trans*-ρ-Menth-2-en-1-ol	1136	–	–	0.42	0.20
32	(*Z*)-Tagetone	1147	–	–	–	0.33
33	Borneol	1165	1.52	1.38	5.61	6.50
34	Terpinen-4-ol	1174	2.60	1.03	10.56	4.96
35	α-Terpineol	1186	1.06	–	1.26	–
36	γ-Terpineol	1199	–	3.04	–	0.68
37	ρ-Cymen-8-ol	1179	–	–	0.94	0.32
38	Thymol methyl ether	1233	3.38	–	1.67	1.63
39	Neral	1235	–	1.35	–	–
40	Carvacrol methyl ether	1241	–	–	2.25	2.48
41	Isobornyl acetate	1283	0.07	–	0.27	0.64
42	Thymol	1288	16.32	–	0.10	–
43	Bornyl acetate	1289	0.08	0.22	–	–
44	Carvacrol ethyl ether	1297	–	–	23.32	31.80
45	Carvacrol	1298	–	0.30	2.89	tr
46	ρ-vinyl guaiacol	1307	23.55	–	–	3.97
47	Dihydro citronellol acetate	1319	–	–	–	0.27
48	Thymol acetate	1345	0.09	–	0.48	–
49	4′-Methoxy-Acetophenone	1346	–	–	–	1.32
50	Eugenol	1354	–	0.08	–	1.24
51	Neryl acetate	1361	–	0.18	–	–
52	Carvacrol acetate	1370	–	–	tr	0.15
53	α-Ylangene	1372	tr	–	–	0.10
54	β-Bourbonene	1387	0.08	tr	tr	0.18
55	(*E*)-Caryophyllene	1416	1.91	–	2.04	4.27
56	(*E*)-Caryophylene	1418	–	3.02	–	–
57	β-Cedrene	1419	–	–	–	0.24
58	β-Copaene	1430	tr	0.09	tr	0.30
59	Aromadendrene	1439	0.15	tr	tr	–
60	α-Humulene	1450	0.09	–	0.11	0.34
61	9-*epi*-(*E*)-Caryophyllene	1464	–	0.15	–	–
62	Dauca-5,8-diene	1470	–	tr	0.06	0.35
63	*trans*-Cadina-1(6),4-diene	1475	–	–	–	0.15
64	γ-Gurjunene	1476	0.15	0.36	–	–
65	Viridiflorene	1496	0.10	–	–	–
66	Bicyclogermacrene	1500	0.18	–	–	–
67	β-Bisabolene	1504	2.84	2.36	1.03	1.12
68	δ-Amorphene	1513	–	0.08	–	0.40
69	γ-Cadinene	1514	0.16	–	–	–
70	Gerany butanoate	1562	–	0.25	–	–
71	Spathulene	1577	0.31	–	–	–
72	Spathulenol	1577	–	–	–	1.41
73	Caryophyllene oxide	1582	0.47	–	5.93	3.40
74	β-Copaen-4-α-ol	1590	–	0.36	–	–
75	Viridi florol	1592	–	–	–	0.13
76	Humulene epoxide II	1608	–	–	0.15	0.10
77	α-Acorenol	1632	–	–	–	0.13
78	Caryophylla-4(12),8(13)-dien-5β-ol	1639	–	–	–	0.24
79	α-Eudesmol	1652	–	–	0.19	–
	Monoterpene hydrocarbons		29.88	1.29	26.64	5.70
	Oxygenated monoterpenes		17.09	22.09	27.89	23.16
	Sesquiterpene hydrocarbons		5.97	6.06	3.24	7.45
	Oxygenated sesquiterpenes		0.47	0.36	6.27	5.41
	Phenolic monoterpenes		16.32	0.38	2.99	1.24
	others		28.13	1.70	28.44	41.79
Those composition possessing less than 0.1%						
80	Tricyclene	921	tr	–	tr	tr
81	Thuja-2,4(10)-diene	954	–	–	tr	tr
82	Sabinene	969	–	–	0.07	tr
83	*trans*-meta-Mentha-2,8-diene	979	–	0.08	–	–
84	Dehydroxy-trans-Linalool oxide	991	–	0.06	–	–
85	α-Phellandrene	1002	–	–	–	0.06
86	δ-3-Carene	1008	–	–	–	0.02
87	σ-3-Carene	1009	0.06	–	–	
88	(*E*)-β-Ocimene	1044	–	0.07	–	–
89	*p*-Mentha-2,4(8)-diene	1085	0.05	–	–	–
90	ρ-Mentha-2,4(8)-diene	1087	0.05	tr	–	–
91	α-Campholenal	1122	–	–	0.01	–
92	*trans*-Sabinol	1137	–	–	–	0.06
93	ρ-Menth-3-en-8-ol	1145	–	–	–	0.09
94	Nerol oxide	1153		0.07	–	–
95	σ-Terpineol	1162	0.01	–	–	–
96	Cumin aldehyde	1238	–	–	0.02	–
97	*cis*-Piperitone epoxide	1250	–	–	0.02	–
98	*n*-Tridecane	1300	–	–	0.03	–
99	σ-Elemene	1335	0.08	–	–	–
100	α-Copaene	1374	–	–	0.01	–
101	Modheph-2-ene	1382	–	–	0.02	–
102	β-Elemene	1386	–	0.07	tr	–
103	α-Isocomene	1387	–	–	0.05	–
104	β-Isocomene	1407	–	–	0.03	–
105	α-trans-Bergamotene	1430	tr	tr	tr	–
106	allo-Aromadendrene	1458	tr	tr	–	–
107	*cis*-Cadina-1(16),4-diene	1463	–	0.02	–	–
108	*cis*-Muurola-4(14),5-diene	1465	–	–	0.05	–
109	4,5-di-*epi*-Aristolochene	1471	–	–	–	0.02
110	γ-Muurolene	1478	–	–	–	0.06
111	ar-Curcumene	1479	–	–	0.03	–
112	β-Selinene	1490	–	–	tr	tr
113	γ-Amorphene	1494	–	0.03	–	–
114	Pseudowiddrene	1498	0.02	–	–	–
115	*cis*-β-Guaiene	1492	–	–	0.02	–
116	*n*-Pentadecane	1500	–	–	0.03	–
117	β-Sesquiphellandrene	1521	–	0.03	–	–
118	(*E*)*-iso*-γ-Bisabolene	1528	–	0.02	–	–
119	*trans*-Cadina-1,4-diene	1533	tr	–	–	0.05
120	α-Cadinene	1537	tr	–	–	0.07
121	α-Calacorene	1544	–	–	0.01	0.05
122	*trans*-Danca-4(11),7-diene	1556	–	0.02	–	–
123	β-Calacorene	1564	–	–	–	0.09
124	Guaiol	1600	–	0.07	–	–
125	Junenol	1618	–	–	–	0.04
126	1-*epi*-Cubenol	1627	–	–	–	0.07
127	Eremoligenol	1628	–	–	0.07	–
128	*epi*-α-Cadinol	1638	–	–	–	0.07
129	*epi*-α-Muurolol	1640	–	–	–	0.04
130	α-Cadinol	1653	0.01	–	–	–

### The common compounds of thyme oil from the four regions

Among the EO components with the content greater than 0.1%, 1,8-cineole, linalool, terpinen-4-ol, γ-terpinene, borneol, β-bisabolene and α-pinene were the common ingredients in the thyme oils from the four regions.

The content of 1,8-cineole (1.84%–10.16%), which is the highest in the QY-thyme oil (10.16%), was similar to that from *T*. *vulgaris* L. with a percentage of 9.8%^23^. Furthermore, 1,8-cineole has been widely used in perfume industry and pharmaceutical industry^24^. Compared to the essential oil of thyme (*T*. *vulgaris* L. and *T*. *zygis* L.) in International Standard (ISO 19,817: 2017), the percentage of linalool (1.22%–12.80%) and terpinen-4-ol (1.03%–10.56%) in this study were far in excess of this standard. Meanwhile, the content of γ-terpinene (0.15%–11.11%) and α-pinene (0.07%–1.04%) were in accordance with International Standard (ISO 19,817: 2017).

The borneol was observed in amounts of 6.50% in YL-thyme oil, similar to that of JB-thyme oil (5.61%). It showed the content of 1.52% in the QY-thyme oil, similar to that of LD-thyme oil (1.38%). Nowadays, it is a famous compound in prescription for cardiovascular and cerebrovascular diseases^25^. Additionally, β-bisabolene is a popular resource for the synthesis of many natural products and it has been approved as a food additive in Europe^26^. The content of β-bisabolene was the highest in QY-thyme oil (2.84%), followed by that of LD-thyme oil (2.36%), JB-thyme oil (1.03%) and YL-thyme oil (1.12%).

### The individual thyme oil composition from the four regions

Notably, the carvacrol ethyl ether was firstly discovered as a new natural product and its amount is significant in YL-thyme oil (31.80%) and JB-thyme oil (23.32%). It is the pale yellow clear oily liquid and has spicy odor. Carvacrol ethyl ether has been used as a synthetic flavoring substance with no safety concern at current levels of intake evaluated by the Joint FAO/WHO Expert Committee on Food Additives (JECFA). The interesting component ρ-vinyl guaiacol was indicated with the highest content in QY-thyme oil (23.55%), followed by that of YL-thyme oil (3.97%). Vinyl guaiacol appeared as colorless or canary yellow liquid that mostly found in the volatile from corn alcohol fermentation and it is a high value-added product widely used in cosmetic, pharmaceutical and chemical industries^27^. Additionally, JB-thyme oil displayed the highest content of terpinen-4-ol (10.56%) while 4.96% in YL-thyme oil. Thymol, the isomeric of carvacrol, was observed as a dominant compound (16.32%) in QY-thyme oil. It exhibited significantly antifungal, antibacterial and anti-inflammatory properties, together with anticonvulsant and antiepileptogenic activities as adjuvant to AEDs^28^. Finally, there were two components that had not been identified. One compound (RT = 16.32, RI = 1,277.300) composed 54% of the total volatile ingredients in LD-thyme oil. And another one (RT = 8.6, RI = 1,027.931) accounted for 10.16% of the volatile composition in YL-thyme oil.

### Multivariate analysis

The content of compounds greater than 0.1% of the thyme oils had been used for chemometric analysis. Both the chemical composition and their content of the total EOs from the four regions were obviously different. It was interesting that the thyme oils from JB and YL were in great similarity. It might result from the close distance between JB and YL, as well as the similar growing conditions such as climate, strength of illumination, humidity of soil and air etc. Thus, growing condition might be the main influencing factor to the chemo-variation in the thyme oils produced from the four regions.

### PCA and HCA analyses of the thyme oil compounds with content greater than 0.1%

**Figure 4:**
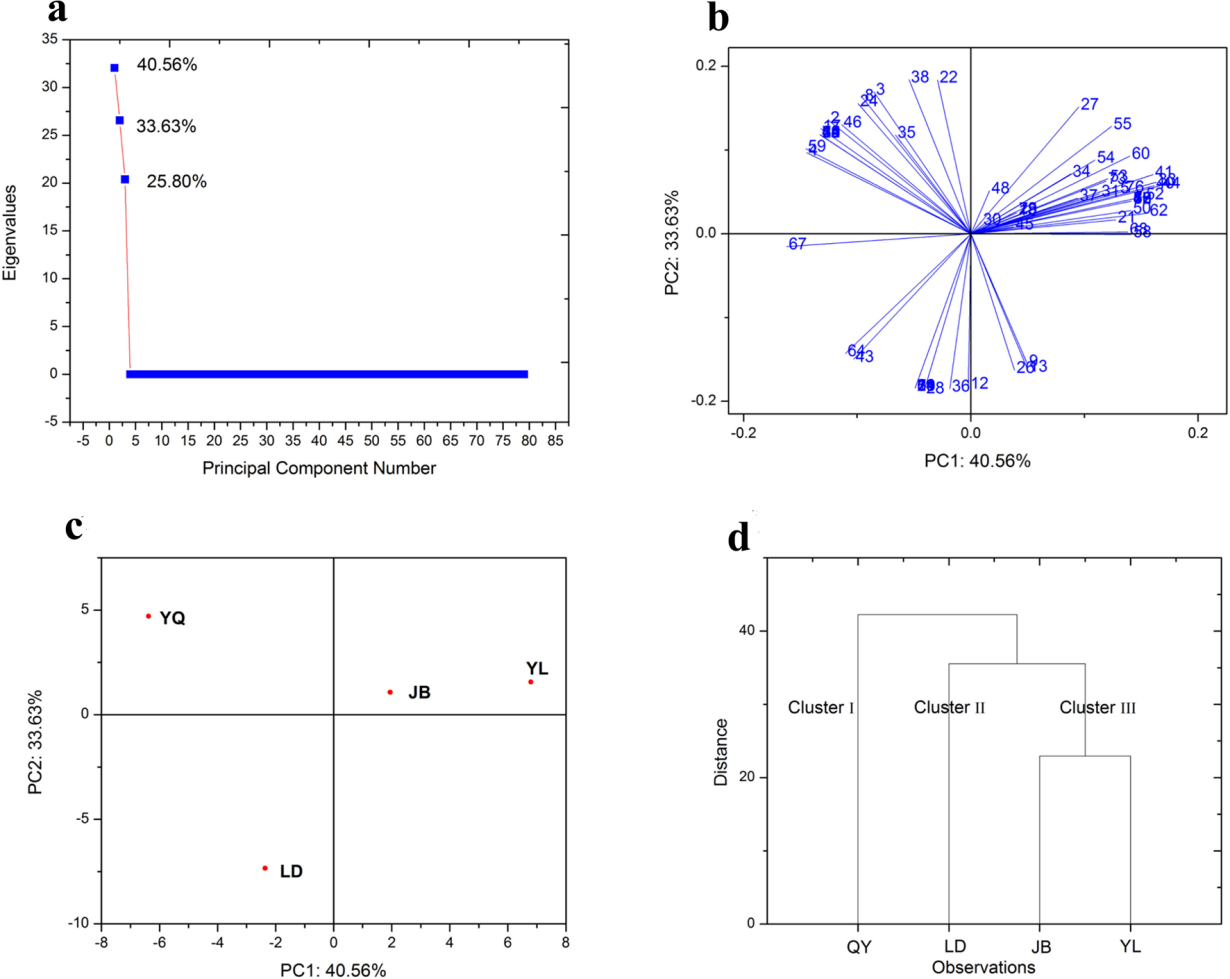
PCA and HCA plots of the thyme oil compositions from the four regions: eigenvalues of the correlation matrix (**a**); the loading plot of active chemical compounds (**b**); the score plot of the tested thyme oil (**c**); the corresponding dendogram (**d**).

PCA is an unsupervised multivariate method and transforms the original set of variables to a new set of uncorrelated variables called principal components (PCs). By plotting the PCA scores, it is possible to visually assess similarities between samples and determine whether samples can be grouped or not^29^. In present study, score plots were created in order to assess clustering tendency of samples, and three significant principal components (PCs) explained 40.56% (PC1), 33.63% (PC2) and 25.80% (PC3) of total variance (Fig. 4a). As shown in Fig. 4c, YL-thyme oil had relatively high scores on PC1, while the QY-thyme oil with the mostly negative PC1 scores. On PC2, the LD-thyme oils had quite negative scores.

The loading vectors responsible for the separations were distributed as Fig. 4b: loading 33, 41, 40 and 44, corresponding to compounds borneol, isobornyl acetate, carvacrol methyl ether and carvacrol ethyl ether, respectively. They were principally responsible for the discrimination of the YL-thyme oil on the PC1. The vectors 28 and 36, represented by linalool and γ-terpineol, were principally involved in the discrimination on the LD-thyme oil. And the vector 42 (thymol) and 46 (ρ-vinyl guaiacol) were strongly featured in separating QY-thyme oil with high negative scores on the PC1.

As with PCA, HCA is also an unsupervised multivariate method, which evaluates the samples by taking account of the chosen distance between samples and group average method was adopted when Euclidean Distance was applied as the measurement^30^. Using D_linkage_ = 22.9 as a criterion for the selection of the number of groups, three clusters were obtained: Cluster I (QY-thyme oil), Cluster II (LD-thyme oil) and Cluster III (JB-thyme oil and YL-thyme oil) (Fig. 4d) and the obtained clusters are in complete agreement with the groups previously defined by PCA.

### In vitro antioxidant activities of thyme oils from the four regions

Phytochemicals are known to have a complex nature hence no single assay will accurately reflect all of the radical sources or all antioxidants in a mixed or complex system^31^. In this study, the in vitro antioxidant activity of thyme oils was evaluated using the DPPH, ABTS, FRAP and TBARS methods.

### DPPH assay

**Figure 5:**
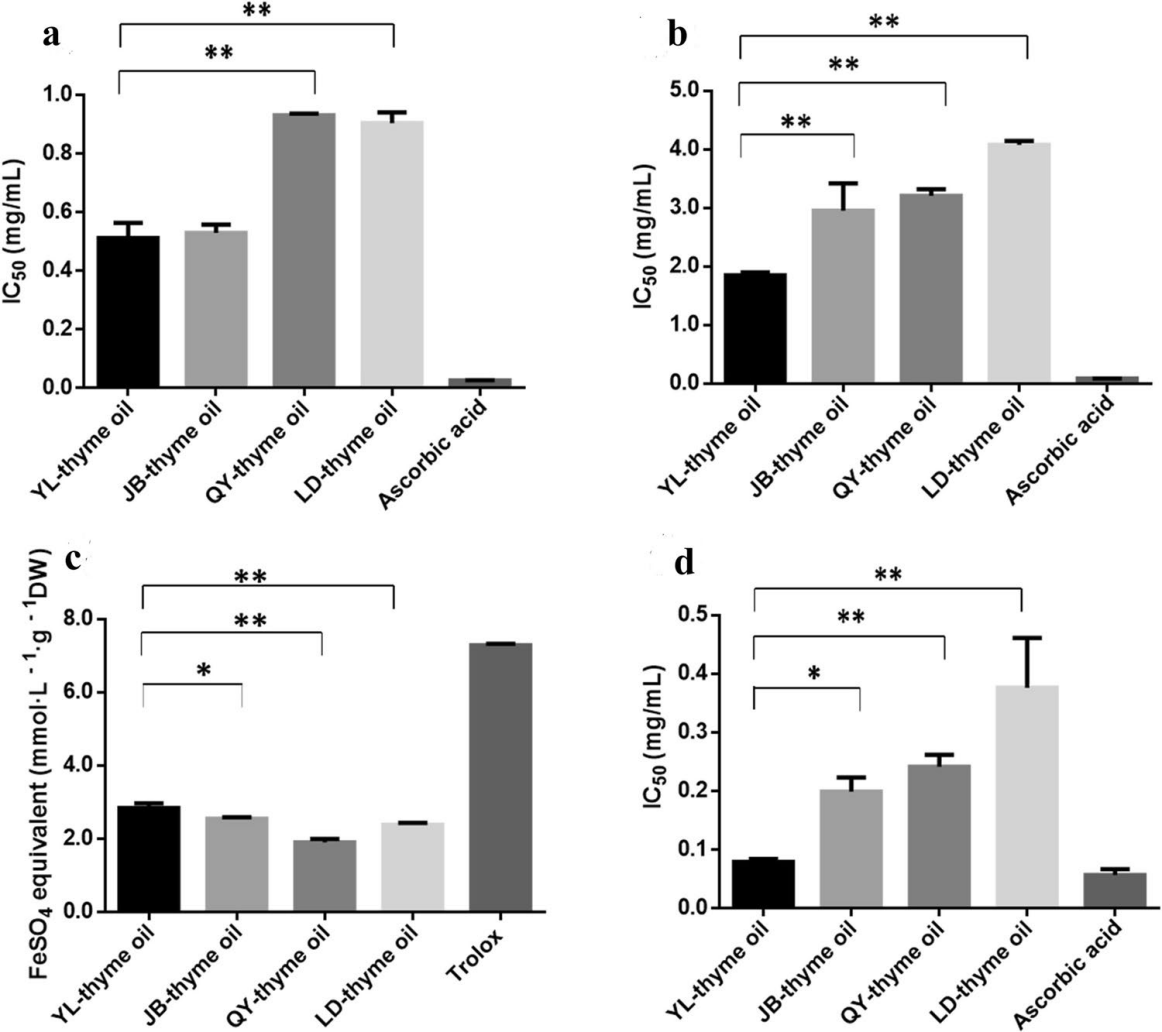
The IC_50_ value of thyme oils on DPPH (**a**), ABTS (**b**) and TBARS (**d**) and FeSO_4_ equivalent on FRAP (**c**). Results are presented as means ± SD (*n* = 3). ** Represents *p*-value less than 0.01 and * represents *p *value less than 0.05.

DPPH molecule that contains a stable free radical has been widely used to evaluate the radical scavenging ability of antioxidants. Comparing with the standard antioxidant ascorbic acid (IC_50_ = 0.026 mg/mL), the YL-thyme oil possessed the highest antioxidant activity (IC_50_ = 0.512 mg/mL), followed by 0.530 mg/mL in JB-thyme oil, 0.905 mg/mL in LD-thyme oil and 0.931 mg/mL in QY-thyme oil (Fig. 5a). The results indicate that YL-thyme oil have better potential as radical scavenger antioxidants than other essential oils.

### ABTS assay

ABTS method is more reliable due to solubility of the ABTS reagent in both aqueous and organic solvents and rapid reaction with lipophilic as well as hydrophilic antioxidant species as compared to DPPH^32^. The IC_50_ values of ascorbic acid, YL-thyme oil, JB-thyme oil, QY-thyme oil and LD-thyme oil were 0.088, 1.848, 2.951, 3.205 and 4.077 mg/mL, respectively. The YL-thyme oil exhibited the best antioxidant capacity with the rate of 98.2% at 2.109 mg/mL. These results revealed that the YL-thyme oil possessed the highest antioxidant activity among the four regions (Fig. 5b).

Characterized by being simple, rapid, accurate and highly sensitive^.^, DPPH and ABTS are often combined to determine the activity of EOs^33^. Moreover, the YL-thyme oil and JB-thyme oil showed similar DPPH and ABTS radical scavenging activity. These results were consistent with the multivariate statistical analyses (PCA and HCA), which indicated that chemical compositions of thyme oils greatly affected the antioxidant activity.

### FRAP assay

FRAP assay measures the reducing potential of an antioxidant reacting with a ferric tripyridyltriazine [Fe^3+^-TPTZ] complex and produces a colored ferrous tripyridyltriazine [Fe^2+^-TPTZ], so FRAP is a reasonable screen for the ability to maintain redox status in cells or tissues^31^. Calibration curves (Y = 0.0379X + 0.0103), in the range of 1 mmol·L^−1^–33 mmol·L^−1^, showed good linearity (R^2^ ≥ 0.9992). The reducing power capacities results follow the same pattern as in case of radical scavenging assay, in which the YL-thyme oil was the most active one (2.917 mmol·L^−1^·g^−1^DW), followed by that from JB (2.549 mmol·L^−1^·g^−1^DW), LD (2.413 mmol·L^−1^·g^−1^DW) and QY (1.956 mmol·L^−1^·g^−1^DW). Comparing these results with the standard antioxidant trolox (7.28 mmol·L^−1^·g^−1^DW), it was revealed that the thyme oils had significantly lower reducing potential than the standard (Fig. 5c).

### TBARS assay

Lipid peroxidation is an oxidative alteration of polyunsaturated fatty acids in the cell membranes that generates a number of degradation products. It can be initiated when fatty acids or fatty acyl side chains are attacked by free radicals. This oxidation process could affect the structural and functional damage of biomolecule^34^. For this reason, it is necessary to determine the function of these thyme oils on lipid peroxidation phenomenon. In biological systems, MDA is a reactive species that participates in the cross-linking of DNA with proteins and damages liver cells^35^. In the present study, Fe^2+^- H_2_O_2_ system was used to induce lipid peroxidation in mouse liver homogenate. The lipid peroxidation inhibition effects of thyme oils increased with the increase of sample concentrations and significant differences (*p* < 0.05) among the tested thyme oils were found.

By comparing the IC_50_ values of YL-thyme oils with that of the authentic antioxidant ascorbic acid, it was found that the antioxidant activity of (IC_50_ = 0.080 mg/mL) was quite comparable with that of ascorbic acid (IC_50_ = 0.058 mg/mL). At a concentration of 0.563 mg/mL, the inhibition effect of YL-thyme oil was 88.90%. The others were ranked in the following order: JB-thyme oil (IC_50_ = 0.198 mg/mL), QY-thyme oil (IC_50_ = 0.241 mg/mL) and LD-thyme oil (IC_50_ = 0.376 mg/mL) (Fig. 5d). These results indicate that the thyme oils significantly inhibited MDA formation in the liver tissue and could protect cell membranes from lipid oxidation.

### The correlations of thyme oil components with antioxidant activities

Furthermore, it is important to correlate and understand which compounds contribute to the different antioxidant assays, showing specific antioxidant potential for the different radicals depending on their chemical structure. The correlations between thyme oil components and antioxidant activities were reported in Table 3. It is well known that the smaller the IC_50_ value is, the better the antioxidant activity is. Therefore, compounds present in thyme oils showed a strong and positive correlation with the free radical scavenging activity (DPPH· and ABTS^·+^), FRAP as well as TBARS. In contrast to this finding, β-bisabolene was inversely and strongly related to the DPPH (0.96561, *p* < 0.05). This inverse relationship would indicate that the higher content of the β-bisabolene did not exert protection, but actually promoted oxidation. Interestingly, carvacrol ethyl ether presented statistically significant positive correlations with DPPH (− 0.96372, *p* < 0.05) and the Code of Federal Regulations lists it as a synthetic flavoring substance that could be safely used in foods (21CFR172.515). Carvacrol methyl ether (− 0.95213, *p* < 0.05) and borneol (− 0.95411, *p* < 0.05) both showed statistically significant positive correlation with DPPH. All the compounds mentioned above are on the list of flavoring substances provided by Regulation (EC) No 2232/96 of the European Parliament and of the Council, which was introduced in Annex I to Regulation (EC) No 1334/2008 of the European Parliament and of the Council.

**Table 3:** The correlations between thyme oil components and antioxidant activities. ** and *: significant at the 0.01 and 0.05 probability levels, respectively.

Components	Antioxidant activity			
	DPPH	ABTS	FRAP	TBARS
Borneol	− 0.95708*	− 0.89822	0.83123	− 0.78727
Carvacrol methyl ether	− 0.95469*	− 0.86998	0.82162	− 0.75053
Carvacrol ethyl ether	− 0.96665*	− 0.93093	0.85974	− 0.83460
β-Bisabolene	0.96544*	0.81295	− 0.86444	0.70027
Artemisia triene	− 0.72122	− 0.92462	0.76939	− 0.96586*
α-Terpinne	− 0.72122	− 0.92462	0.76939	− 0.96586*
(Z)-Tagetone	− 0.72122	− 0.92462	0.76939	− 0.96586*
Isobornyl acetate	− 0.88440	− 0.98067*	0.82587	− 0.93715
Dihydrocitronellol acetate	− 0.72122	− 0.92462	0.76939	− 0.96586
4′-Methoxy-Acetophenone	− 0.72122	− 0.92462	0.76939	− 0.96586*
Eugenol	− 0.71360	− 0.91657	0.77973	− 0.96836*
Carvacrol acetate	− 0.79902	− 0.96479*	0.81926	− 0.98119*
β-Cedrene	− 0.72122	− 0.92462	0.76939	− 0.96586*
Dauca-5,8-diene	− 0.81422	− 0.96651*	0.85879	− 0.99426*
trans-Cadina-1(6),4-diene	− 0.72122	− 0.92462	0.76939	− 0.96586*
Spathulenol	− 0.72122	− 0.92462	0.76939	− 0.96586*
Viridiflorol	− 0.72122	− 0.92462	0.76939	− 0.96585
α-Acorenol	− 0.72122	− 0.92462	0.76939	− 0.96586*
Caryophylla-4(12),8(13)-dien-5β-ol	− 0.72122	− 0.92462	0.76939	− 0.96586
δ-Amorphene	− 0.68532	− 0.88631	0.79189	− 0.96065*

The correlation between ABTS and compounds, including isobornyl acetate (− 0.98067, *p* < 0.05), carvacrol acetate (− 0.96479, *p* < 0.05) and dauca-5,8-diene (− 0.96651, *p* < 0.05) was obvious. This tendency is explained by the high scavenging power of these components. Similarly, the latter two were considerably correlated with TBARS (− 0.98119 and − 0.99426, *p* < 0.05, respectively) and these observations are consistent with published data^36^. However, for FRAP, all of them were not statistically significant. These compounds mentioned above were found in the highest amounts in the YL and JB thyme-oil. This indicated that the higher level of these components is likely to be closely related to the relatively high antioxidant activity of YL and JB-thyme oil. These differences observed between assays are related to the individual molecular structure of each compound which indicated that stereoisomerism, functional groups distribution and any other structural parameters such as the oxidation state of the C ring, the hydroxylation and methylation pattern also are expected to affect the final value. In particular, there were good correlations of carvacrol ethyl ether both with IC_50_ value of DPPH and ABTS and carvacrol acetate both with IC_50_ value of ABTS and TBARS, which could be recommended as an indicative component for the antioxidant capacity. It is acknowledged that carvacrol is used to defined the quality of the *Thymus* species and widely used in the food industry as antimicrobial and antioxidant agent. Thus, in the present work, carvacrol methyl ether, carvacrol ethyl ether and carvacrol acetate were all found to be significantly associated with antioxidant activity, implying the carvacrol analogues as potential natural antioxidants of thyme oils from Loess Plateau in China.

### In vivo antioxidant activity of thyme oil on the zebrafish

Although in vitro assays are used for rapid screening but their results cannot be directly extrapolated to in vivo conditions. The experimental models are therefore used to simulate in vivo conditions. Hence, we also evaluated the antioxidant capacity of thyme oils using zebrafish model.

### Lethal and teratogenic effects of thyme oil

To determine the toxicity of thyme oils, we investigated the survival rate of zebrafish larvae. The thyme oil had no toxic effect up to 40 μg/mL. Therefore, concentrations of thyme oil less than 40 μg/mL are suitable for the in vivo experiments.

**Figure 6:**
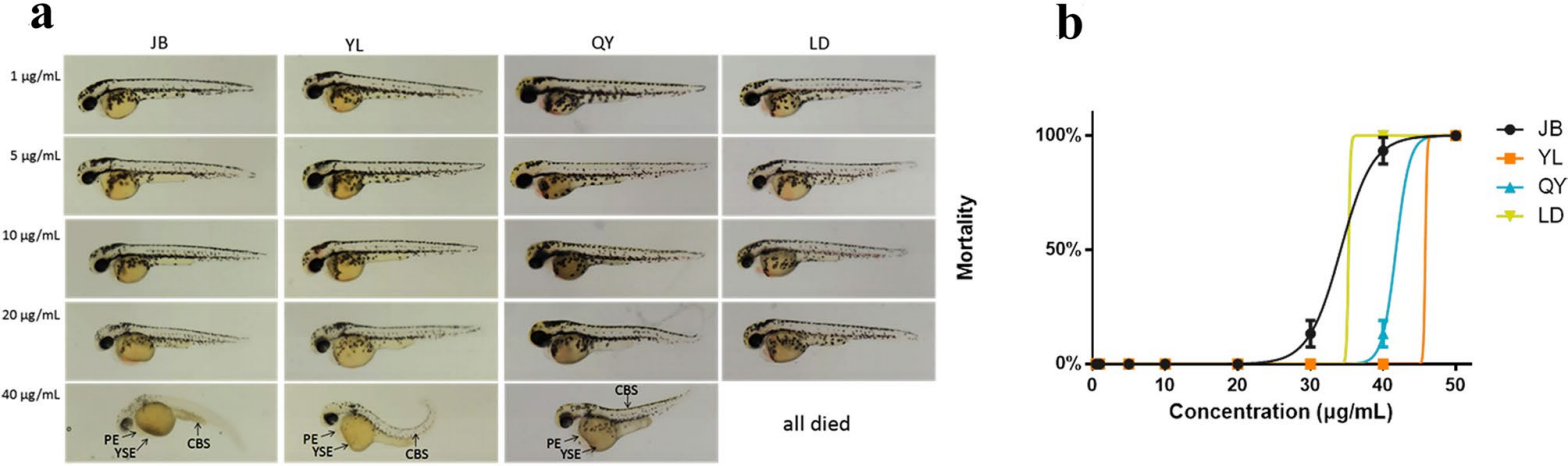
The toxicity of thyme oils in zebrafish larvae (**a**). Mortality curves until 48 hpf (**b**). Results are presented as means ± SD (*n* = 15). YSE: yolk sac edema; PE: pericardial edema; CBS: curved body shape.

When the concentration reached 40 μg/mL, these larvae treated with the thyme oils at 48 hpf from four producing areas showed different degrees of morphological abnormalities, including yolk sac edema, pericardial edema, and curved body shape (Fig. 6a). Pericardial edema and yolk sac edema were the most pronounced morphological alterations. Pericardial edema was first observed in the LD-thyme oil and all zebrafish larvae in this group were dead at the concentration of 40 μg/mL. The curved body shape was first observed in the QY-thyme oil at the concentration of 40 μg/mL.

Figure 6b shows the lethal effects of thyme oil at 48 hpf and mortality rates in the treatment group exhibited a dose and time-dependent increase. The values of the 1/3 LC_1_, LC_1_ and LC_10_ of the four thyme oils were determined to be around 5, 10 and 20 μg/mL.

### Antioxidant activity in Tg (krt4: NTR-hKikGR)^cy17^ zebrafish larvae

**Figure 7:**
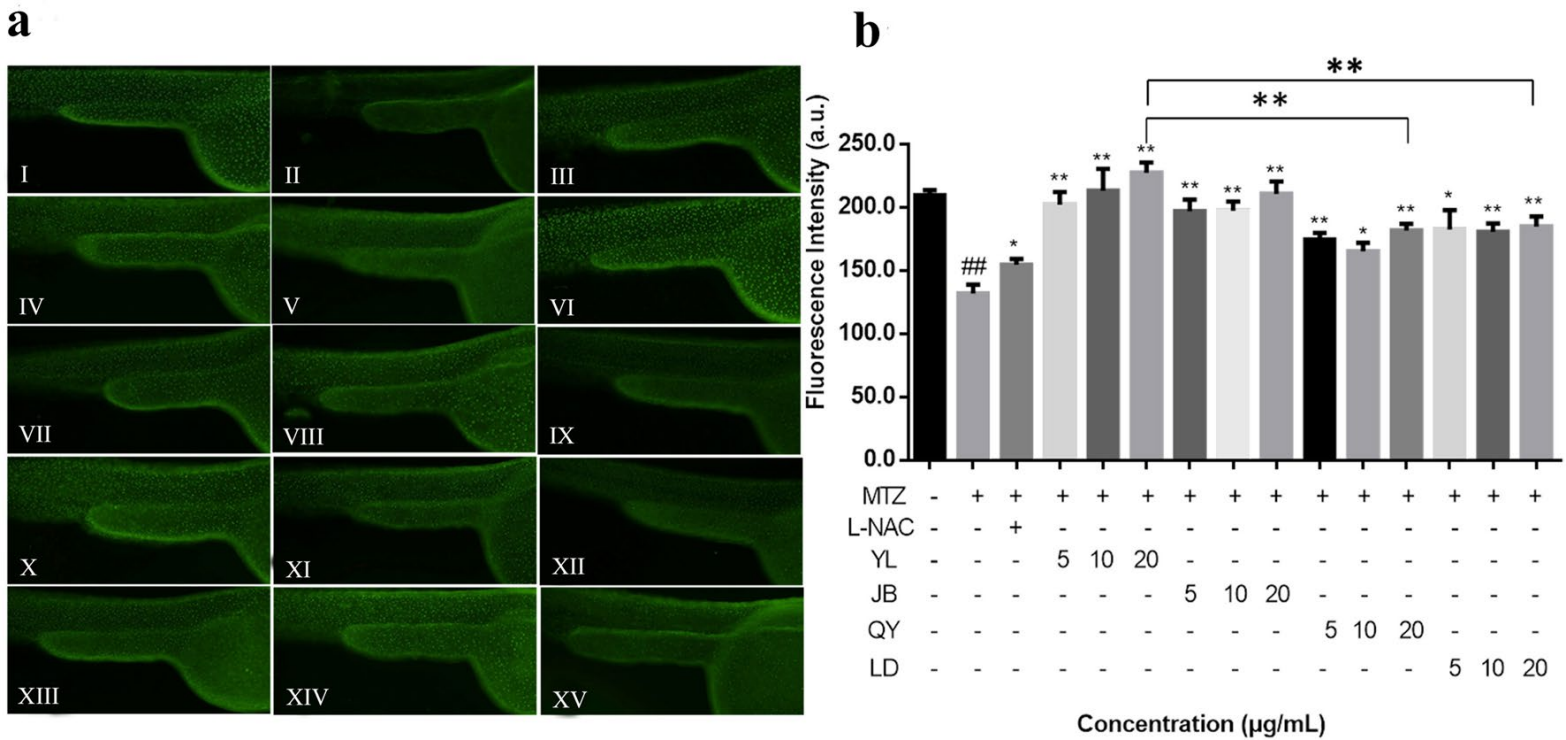
Thyme oils inhibited MTZ-induced oxidative insults in skin cells of Tg (krt4: NTR-hKikGR)^cy17^ transgenic zebrafish (**a**): Control group (I); MTZ-treatment group (II); MTZ + L-NAC-treatment gruop (III); MTZ + YL-thyme oil-treatment group at the concentration of 5 μg/mL (IV), 10 μg/mL (V) and 20 μg/mL (VI); MTZ + JB-thyme oil-treatment group at the concentration of 5 μg/mL (VII), 10 μg/mL (VIII) and 20 μg/mL (IX); MTZ + QY-thyme oil-treatment group at the concentration of 5 μg/mL (X), 10 μg/mL (XI) and 20 μg/mL (XII); MTZ + LD-thyme oil-treatment group at the concentration of 5 μg/mL (XIII), 10 μg/mL (XIV) and 20 μg/mL (XV); Determination of fluorescence intensity by Image analysis (b); The values are expressed as mean ± SD (*n* = 15). * Represents *p*-value less than 0.05 and ** represents *p*-value less than 0.01 vs. MTZ group; # represents *p *value less than 0.05 and ## represents *p *value less than 0.01 vs. control group.

The in vivo capability of thyme oils on inhibiting oxidative stress was further evaluated using MTZ-induced oxidative insult in Tg (krt4: NTR-hKikGR)^cy17^ transgenic zebrafish model. A transgenic line of Tg (krt4:NTR-hKikGR)^cy17^ is established which overexpresses the NTR-hKikGR fusion protein under the control of the skin-specific krt4 promoter. It provides as a unique quantitative and fast tool to study the signaling molecules which modulate skin apoptosis in living animals^37^. The fluorescence spots of zebrafish are shown in Fig. 7a. and the anti-oxidation results of the thyme oil on transgenic zebrafish treated with MTZ are shown in Fig. 7b. The results showed that the number of fluorescence spots on the epidermal cells of the zebrafish was significantly decreased after the addition of MTZ. In the treatment of different concentrations of thyme oils (5, 10 and 20 μg/mL), the number of fluorescence spots on the epidermal cells increased significantly compared with the MTZ-treatment group, indicating that the thyme oils can resist the overproduction of ROS and skin cell apoptosis induced by MTZ. The antioxidant capacity of the thyme oils is better than that of the L-NAC-treatment group, especially the antioxidant capacity of YL-thyme oil is the most prominent, followed by that from JB-thyme oil, QY-thyme oil and LD-thyme oil.

### Protective effects of thyme oil on ROS generation in AAPH-induced oxidative stress

The antioxidative effect of thyme oil on ROS production in zebrafish larvae was examined by detection of DCFDA. Fluorescent photographs of larvae showed that the untreated zebrafish had low fluorescence intensity, whereas AAPH-induced group generated a marked increase in the observed fluorescence intensity, indicating that ROS was produced in the presence of AAPH in the zebrafish larvae.

**Figure 8:**
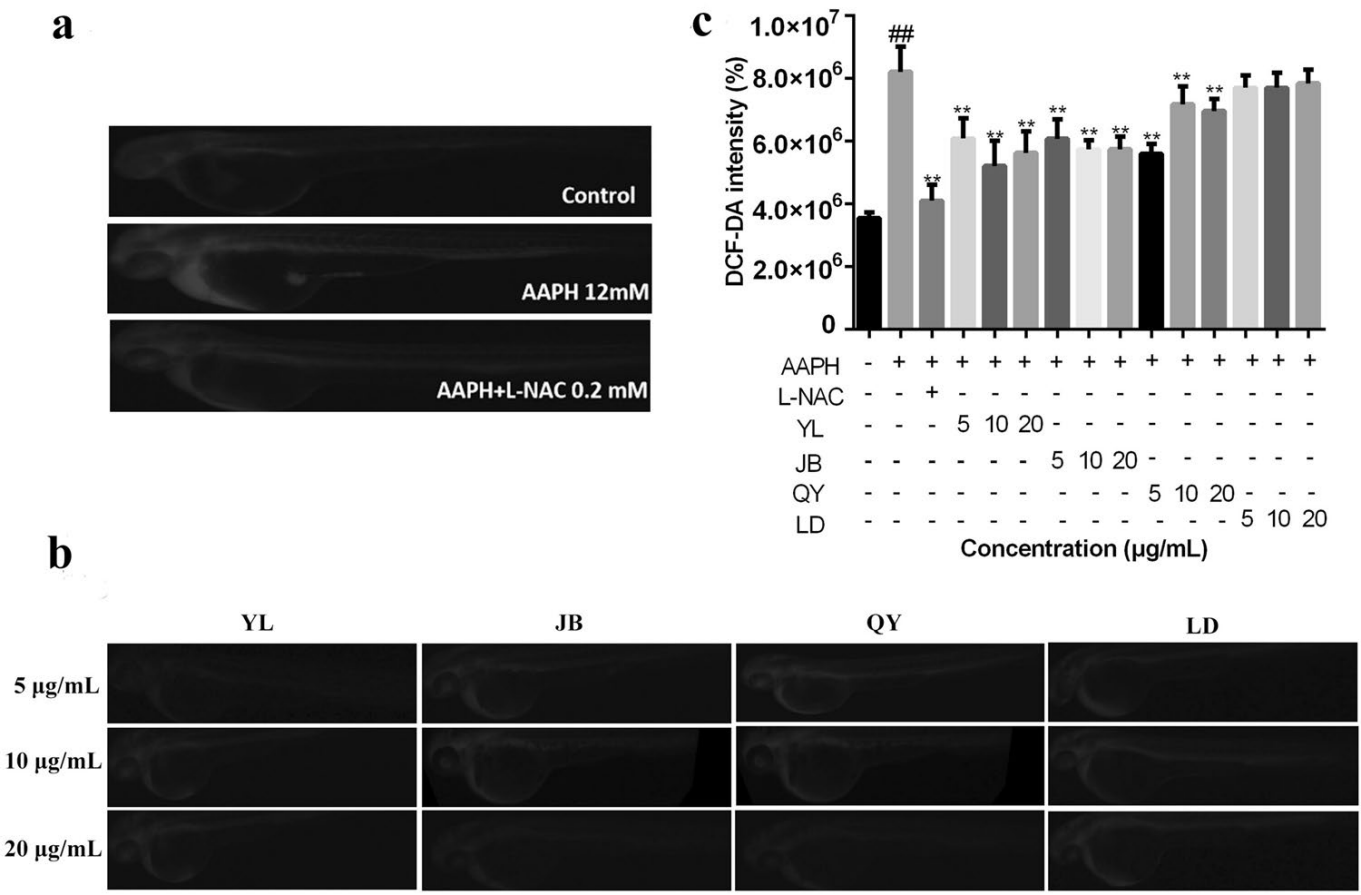
Determination of ROS levels in zebrafish larvae from 48 hpf in Control group, AAPH-treatment group, AAPH + L-NAC-treatment group (**a**); Determination of ROS levels in zebrafish larvae from 48 hpf after treatment with YL-thyme oil, JB-thyme oil, QY-thyme oil and LD-thyme oil (**b**); Determination of mean DCF fluorescence intensity by Image analysis (**c**). The values are expressed as mean ± SD (*n* = 15). * Represents *p*-value less than 0.05 and ** represents *p *value less than 0.01 vs. AAPH-induced group; # represents *p*-value less than 0.05 and ## represents *p*-value less than 0.01 vs. control group.

The scavenging efficacy of the thyme oil on the ROS production in AAPH-induced zebrafish larvae was measured. At 48 hpf, the larvae in the YL-thyme oil-treatment and JB-thyme oil-treatment groups (Fig. 8b) exhibited markedly lower fluorescence intensities than those in the AAPH-treatment group (Fig. 8a), which indicated that ROS significantly decreased after thyme oil exposure. Conversely, fluorescence intensity in QY-thyme oil-treatment and LD-thyme oil-treatment groups (Fig. 8c) did not decrease significantly compared to the AAPH-treatment group. Hence, these results indicate that thyme oil are effective antioxidants in AAPH-induced oxidative stress model.

### Mechanism of YL-thyme oil on the AAPH-induced zebrafish larvae

The outstanding in vitro and in vivo antioxidant activities presented above gave an indisputable confirmation that YL-thyme oil and JB-thyme oil showed better antioxidant activities due to the close distance between two of them. YL-thyme oil exerted the best antioxidant activity among the four regions and further studies are required to elucidate the biological mechanisms and signaling pathways underlying the protective effects of the thyme oil.

### Effects of YL-thyme oil on MDA Levels, SOD and CAT activities in AAPH-induced zebrafish larvae

The antioxidant enzymes, SOD and CAT, work within the cells to remove most of superoxides and peroxides before they react with metal ions to form more reactive free radicals. SOD, the first line of defense against free radicals, converts the superoxide radical to H_2_O_2_ and O_2_ by reduction. The H_2_O_2_ is transformed into water and oxygen by CAT^38^. MDA, an end product of lipid peroxidation, represented the level of lipid peroxidation^39^.

**Figure 9:**
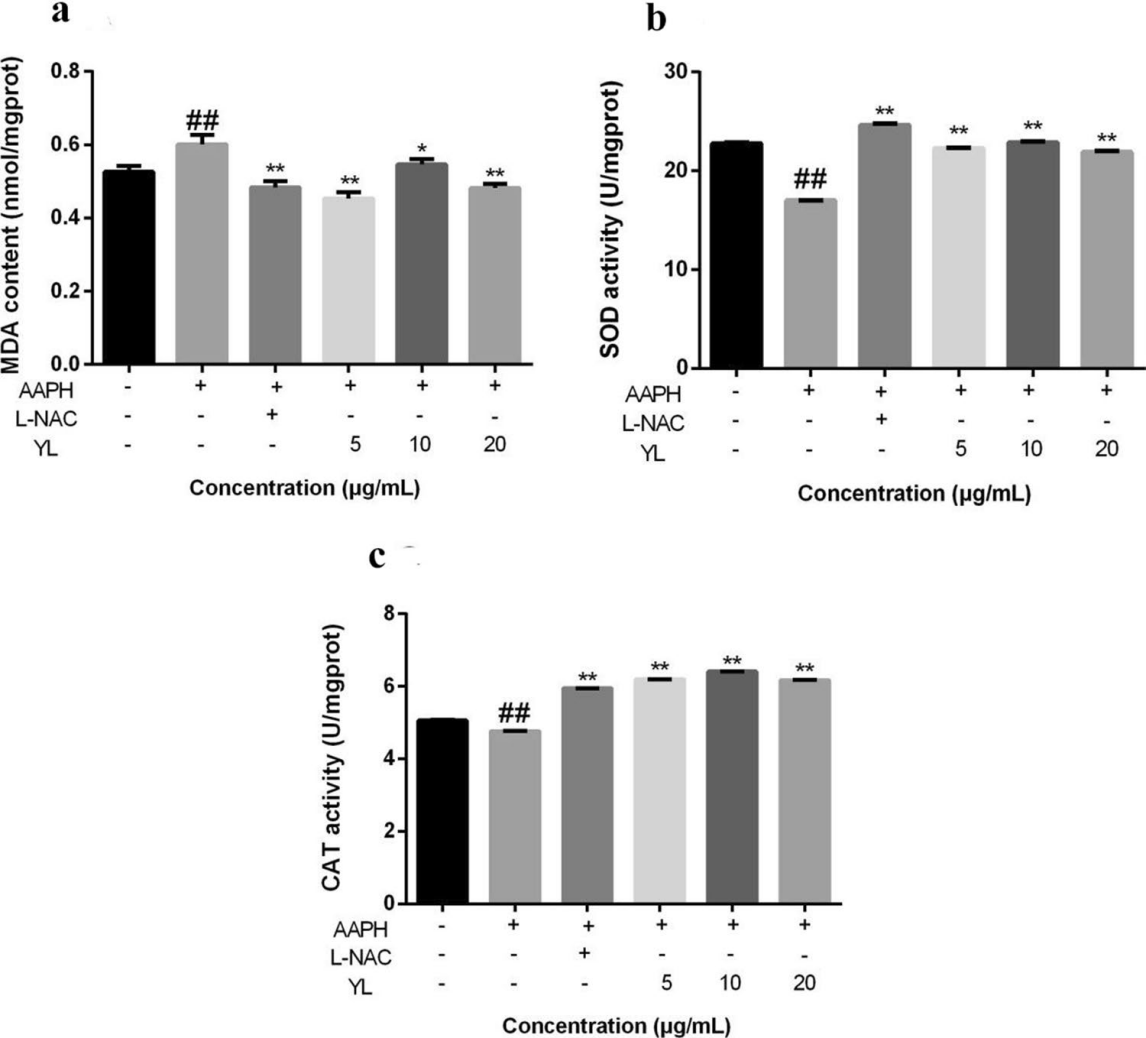
Effects of YL-thyme oil on levels of MDA and antioxidant enzymes activity in AAPH-induced zebrafish larvae. MDA level (**a**), SOD activity (**b**) and CAT activity (**c**) were examined after incubation. The values are expressed as mean ± SD (*n* = 3). * Represents *p*-value less than 0.05 and ** represents *p*-value less than 0.01 vs. AAPH-induced group; # represents *p*-value less than 0.05 and ## represents *p *value less than 0.01 vs. control group.

The MDA levels and antioxidant enzymes activities (SOD and CAT) were examined and shown in Fig. 9. AAPH-induction decreased the activities of SOD, and CAT in zebrafish and increased MDA levels when compared to control group, whereas AAPH-induced zebrafish protected by YL-thyme oil showed remarkable damage prevention. After being treated with YL-thyme oil (5 μg/mL), the activities of SOD and CAT were increased by 1.30 folds, and MDA level decreased by 75% as compared with those of AAPH-induced group. The results implied that the protective effect of YL-thyme oil against oxidative stress might be rationalized because of the impairment of antioxidant defenses system. Obviously, AAPH damaged the antioxidant defense system and promoted the lipid peroxidation by reducing the activities of the antioxidation enzymes and upregulating the formation of MDA in zebrafish, while YL-thyme oil could prevent these harms.

### Effects of YL-thyme oil on gene expression

**Figure 10:**
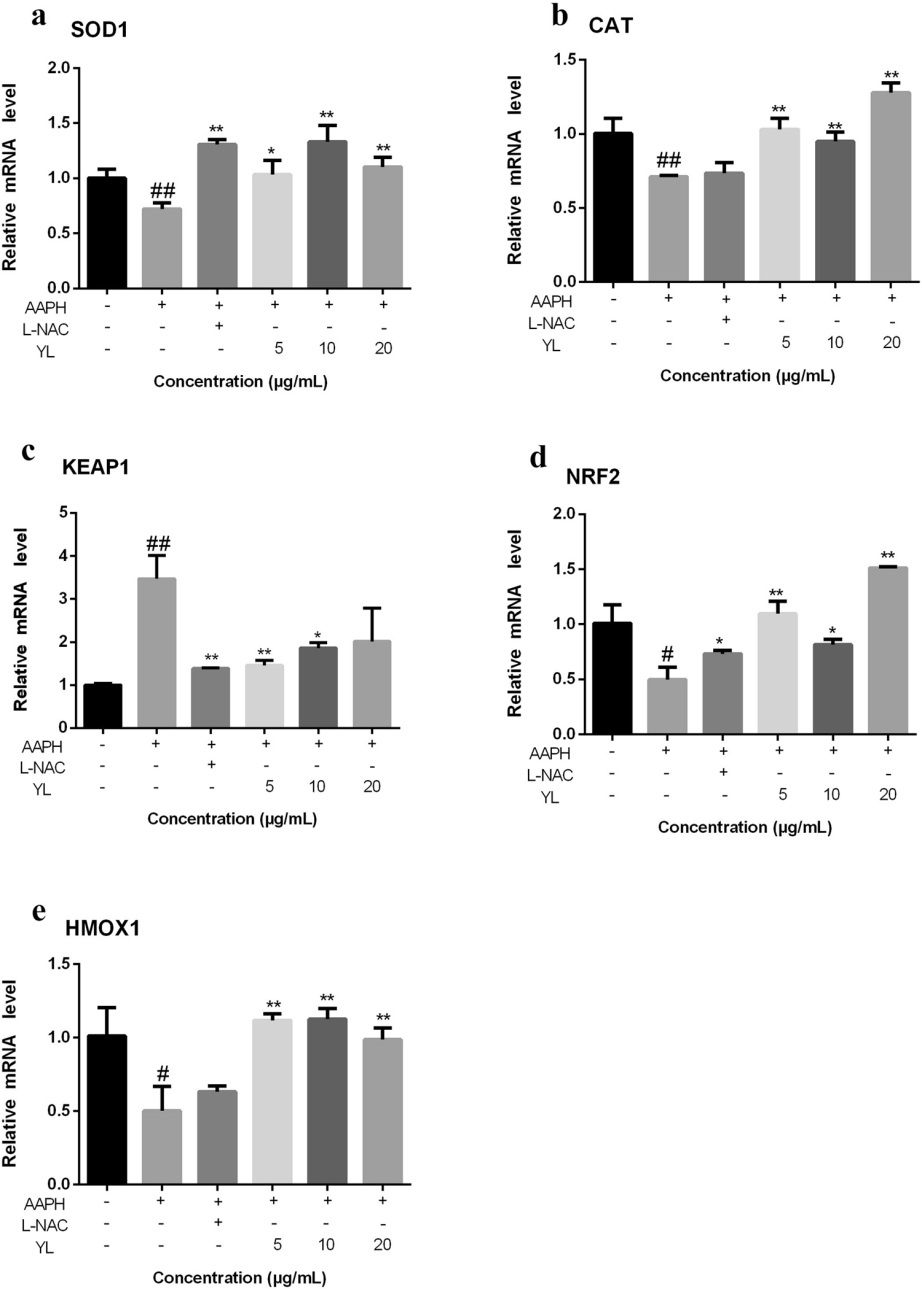
Effects of thyme oil gene expression in zebrafish larvae at 48 hpf. The mRNA levels of Sod1 (**a**), Cat (**b**), Keap1 (**c**) and Hmox1 (**d**) on YL-thyme oil. The values are expressed as mean ± SD (*n* = 3). * Represents *p*-value less than 0.05 and ** represents *p *value less than 0.01 vs. AAPH-induced group; # represents *p *value less than 0.05 and ## represents *p*-value less than 0.01 vs. control group.

In order to investigate the mechanism of YL-thyme oil-treated antioxidant activity, the mRNA expression levels of genes related to the antioxidant activities were measured (Fig. 10). Nrf2 and its endogenous inhibitor, Keap1, function as a ubiquitous, evolutionarily conserved intracellular defense mechanism to counteract oxidative stress and the Keap1/Nrf2 signaling pathway is an important regulatory component of cells in a steady-state environment, protecting cells from oxidative stress^5^. Moreover, Nrf2/Hmox1 pathway is widely studied in vertebrates and play a pivotal role in neurodegenerative disorders. Hmox1, as Nrf2-dependent gene, provides cytoprotective effect and play a crucial role in the development of oxidative and age-related disorders^40^. Also, Hmox1 is known to play an important role in cellular protection against oxidative insult in cardiovascular disease, including diabetes, and in the alleviation of vascular diseases and various chemical reagents were known to facilitate antioxidant response via activating Hmox1 expression^41^. In the present study, YL-thyme oil-treated zebrafish larvae exhibited significant downregulation of Keap1 expression and upregulation of Nrf2 expression; furthermore, the expression level of Sod1, Cat and Hmox1 were significantly increased in the treatment of YL-thyme oil groups. AAPH-induction decreased the expression of Sod1, Cat, Nrf2 and Hmox1 in zebrafish by 1.39, 1.41, 2.03 and 2.02 folds, respectively and increased Keap1 levels by 3.47 folds when compared to control group. In the YL-thyme oil-treatment group and L-NAC-treatment group, the expression levels of genes encoding the Sod1 (Fig. 10a) and Nrf2 (Fig. 10d) were significantly increased relative to that in the AAPH-treatment groups. The expression levels of genes encoding Cat (Fig. 10b) and Hmox1 (Fig. 10e) were significantly increased relative to the AAPH-treatment group; however, no obvious changes were detected in the L-NAC-treatment groups relative to the control groups. In contrast, the expression levels of genes encoding Keap1 was downregulated in the YL-thyme oil-treatment group (Fig. 10c). Taken together, our data demonstrated that thyme oil might exert the antioxidant activity in AAPH-induced oxidative stress model by activating the cellular Nrf2/Keap1 pathway.

## Conclusions

This is the first study on the chemical constituents, antioxidant activities and biological mechanism of EOs of *T*. *quinquecostatus*. One new natural compound, carvacrol ethyl ether, was firstly discovered in the thyme oils. It has been used as a synthetic flavoring substance with no safety concern. The thyme oils from the four regions were classified into three groups by chemometric analysis, out of which the borneol, isobornyl acetate, carvacrol methyl ether, ρ-vinyl guaiacol, thymol, carvacrol ethyl ether and linalool contributed the most to the classification, indicating that these compounds may be used for the geographical markers of the thyme oils. The YL-thyme oil was found to produce superior EO yields and possess the strongest anti-oxidant activities in vitro and in vivo, suggesting that domestication of this thyme oil is economical for the food industry. It was also proved with correlation analysis that borneol, carvacrol methyl ether, carvacrol ethyl ether, carvacrol acetate, isobornyl acetate and dauca-5,8-diene could be the characteristic constituents responsible for the antioxidant effect of the thyme oils. This indicated that the carvacrol analogues might be developed as potential natural antioxidants of thyme oils from Loess Plateau in China. Furthermore, Keap1/Nrf2 pathway contributed to the anti-oxidant processes of YL-thyme oil. Taken together, our results suggest that thyme oil could be useful as the natural antioxidant and have the potentiality of enhancing the protective effect against oxidative stress. The findings of the present study can provide new insights for enhancing the valuable components and antioxidant capacity of this plant in their EOs. Therefore, this study could lay foundation for the applications of thyme oils in food, pharmaceutical and cosmetic industry, and also provide promising sources for natural antioxidants and a new possible breakthrough point for further bioactivity studies of thyme. Admittedly, there are limitations to the current study. CC-MS has been used to quantified the thyme oils components and we didn’t examine the data further by GC-FID which might be more precise than GC–MS in quantifying complex mixtures. In addition, we cannot completely determine the major contribution of components for the antioxidant activities of thyme oils. The potential antioxidants, especially the carvacrol analogues from the correlation analysis should be assessed in future studies. Furthermore, further studies can be performed to delineate other candidate genes and signaling pathways, especially those closely correlated with anti-oxidation, in thyme oils-mediated effects.

### Ethics statement

All experimental procedures were carried out in accordance with the National Institute of Health Guide for the Care and Use of Laboratory Animals and approved by the Experimental Animal Ethics Committee of the Academic Committee of Beijing University of Chinese Medicine.
